# Contrast-free ultrasound imaging for blood flow assessment of the lower limb in patients with peripheral arterial disease: a feasibility study

**DOI:** 10.1038/s41598-023-38576-x

**Published:** 2023-07-13

**Authors:** Soroosh Sabeti, Rohit Nayak, Robert D. McBane, Mostafa Fatemi, Azra Alizad

**Affiliations:** 1 Department of Physiology and Biomedical Engineering, Mayo Clinic College of Medicine and Science, Rochester, MN USA; 2 Department of Radiology, Mayo Clinic College of Medicine and Science, 200 1st Street SW, Rochester, MN 55905 USA; 3 Department of Cardiovascular, Division of Vascular Medicine, Mayo Clinic College of Medicine and Science, Rochester, MN USA

**Keywords:** Diseases, Peripheral vascular disease, Engineering, Biomedical engineering

## Abstract

While being a relatively prevalent condition particularly among aging patients, peripheral arterial disease (PAD) of lower extremities commonly goes undetected or misdiagnosed due to its symptoms being nonspecific. Additionally, progression of PAD in the absence of timely intervention can lead to dire consequences. Therefore, development of non-invasive and affordable diagnostic approaches can be highly beneficial in detection and treatment planning for PAD patients. In this study, we present a contrast-free ultrasound-based quantitative blood flow imaging technique for PAD diagnosis. The method involves monitoring the variations of blood flow in the calf muscle in response to thigh-pressure-cuff-induced occlusion. Four quantitative metrics are introduced for analysis of these variations. These metrics include post-occlusion to baseline flow intensity variation (PBFIV), total response region (TRR), Lag0 response region (L0RR), and Lag4 (and more) response region (L4 + RR). We examine the feasibility of this method through an in vivo study consisting of 14 PAD patients with abnormal ankle-brachial index (ABI) and 8 healthy volunteers. Ultrasound data acquired from 13 legs in the patient group and 13 legs in the healthy group are analyzed. Out of the four utilized metrics, three exhibited significantly different distributions between the two groups (*p*-value < 0.05). More specifically, *p*-values of 0.0015 for PBFIV, 0.0183 for TRR, and 0.0048 for L0RR were obtained. The results of this feasibility study indicate the diagnostic potential of the proposed method for the detection of PAD.

## Introduction

Peripheral arterial disease (PAD) generally refers to a progressive circulation disorder, characterized by narrowing or occlusion of the peripheral arteries^1^. Lower extremity PAD is the most common form of PAD affecting more than 8.5 million Americans and more than 230 million patients worldwide^2,3^. While traditional atherosclerotic risk factors participate in the development of PAD, nicotine addiction with habitual smoking is considered the most prevalent risk for these patients, and diabetes mellitus is likewise an important variable^4,5^. An early and the most common clinical presentation of PAD is intermittent claudication, which involves pain in the lower limb (particularly the calves) mainly induced by physical activities such as walking and subsiding by rest^6,7^. While PAD associated walking impairment adversely influences quality of life for PAD patients, there is an added elevated risk of developing major adverse cardiac events including myocardial infarction, stroke, heart failure and both cardiovascular death and all-cause mortality^8–10^. Beyond intermittent claudication and major adverse cardiac events, severe PAD can lead to critical limb ischemia which if untreated, might result in leg amputation and a high mortality rate as a result^11^. Therefore, early diagnosis of PAD and proper implementation of guideline indorsed risk factor modification is paramount for effective management of these patients^12^.

Customary clinical diagnostic and monitoring methods for peripheral arterial disease include the use of different imaging modalities such as duplex ultrasonography, computed tomography (CT) and magnetic resonance (MR) angiography^13–15^. For disease screening, symptom evaluation, and periodic disease monitoring, a non-invasive evaluation is more appropriate and involves Doppler assessment and measurement of the ankle brachial index (ABI)^16^. ABI values in the range of 0.9 to 1.4 are considered as normal^17,18^. Values less than 0.9 indicate the presence of arterial disease^19^ with values below 0.5 consistent with severe PAD, suggestive of critical limb ischemia^20^. While readily available, easy to perform, and reproducible, the resting ABI assessment may have limited accuracy in conditions such as diabetes mellitus, chronic kidney disease, arterial calcification, isolated iliac arterial disease and in the elderly^21,22^. Furthermore, ABI may be insensitive to microvascular impairment, which may coexist with macrovascular disease in PAD patients^23–26^. Therefore, normal ABI does not rule out PAD and values greater than 1.4, may indicate high stiffness and poorly compressible arteries (mostly due to arterial calcification)^27,28^. As a result, in such cases, the severity of PAD cannot be properly evaluated.

Arteriolar endothelial function, microvascular regulation and responsiveness to specific stimuli are impaired in patients with PAD which may play a central role in disease development and progression^29–32^. The impaired microvascular reactivity expresses the imbalance between vasodilators and vasoconstrictors, such as nitric oxide and endothelin, respectively. One study reported the assessment of microvascular reactivity by hyperemic response to contraction and cuff occlusion using BOLD imaging^33^. Moreover, in response to chronic metabolic demand in PAD, vascular remodeling include increased diameter and density of microcirculations to accommodate the increased blood flow^34^. Studies suggest that progression of the peripheral arterial disease can lead to disruptions in blood distribution and flow in the muscles of the lower extremity, thus creating the conditions that result in intermittent claudication^35–37^. Many of these studies are performed using MR^38–40^ and CT-based imaging methodologies^41,42^.

In recent years, there has been growing interest in investigating the microvascular flow and muscle perfusion in PAD. However, the use of non-invasive imaging modalities for PAD diagnosis, such as duplex ultrasonography, has been limited to the evaluation of macrovascular blood flow^43,44^. Emergence of plane wave ultrasound imaging has shown promise in quantitative high resolution blood flow imaging without the use of contrast agents^45–48^. Several studies have examined the practicability of novel ultrasound microvasculature and perfusion imaging strategies for evaluation of blood flow variations in patients with PAD^49^. While studies using contrast agents have shown promise for assessing slow flow in small vasculature^50–54^, there is a need for more accessible and cost-effective methods that do not require contrast agents.

Monitoring and analysis of the reactive hyperemic response in the lower limb to externally induced occlusion can have diagnostic significance. It is known that a reduction in peak muscle perfusion capacity occurs in response to ischemic provocation tests, such as thigh-cuff occlusion ^55^. We hypothesize that by evaluating the blood flow variations of the lower extremity in response to thigh-cuff occlusion, we can identify PAD-affected legs. In this paper, a novel pressure-based method is proposed for flow imaging that can assess microvascular reactivity using ultrasound. The method measures the changes in microvascular blood flow in the calf muscles in response to a mechanical stimulus, which can be used to assess the function of the microvasculature in the lower extremities. We demonstrate how the measured metrics can provide potential diagnostic information about the conditions of patients in comparison to their ABI evaluations.

## Results

### Response analysis

In this section, we present a comparison of the results of our methodology for a 72-year-old female PAD patient clinically diagnosed with moderate PAD in the left leg and the results for the left leg of a healthy 62-year-old female volunteer. Resting ABI values for the left leg of the PAD patient measured at the dorsalis pedis (DP), and the posterior tibial (PT) arteries are reported as 0.62 and 0.61, respectively.

**Figure 1:**
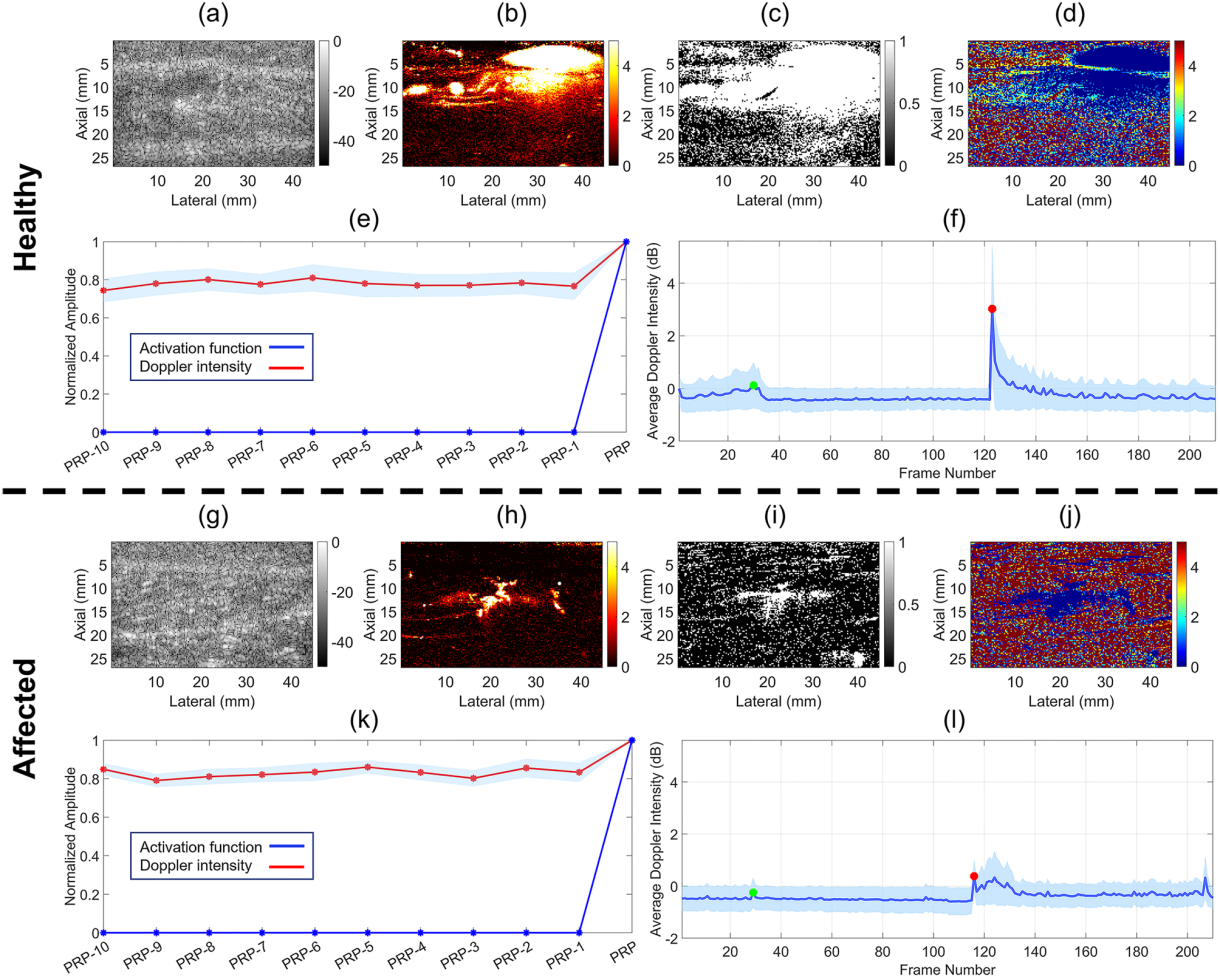
Comparison of the results for the legs of a healthy individual (figures (**a**–**f**), top) and a PAD patient with abnormal ABI (figures (**g**–**l**), bottom). (**a**, **g**) B-modes of the scanned region. (**b**, **h**) Doppler images post-occlusion (**c**, **i**) Binarized post-occlusion correlation masks using a 0.5 threshold on the correlation value. (**d**, **j**) Lag images. (**e**, **k**) Normalized average of Doppler Intensity variations (red) of all pixels within correlation masks (shaded light blue region depicts half of the standard deviation on each side of the plot) and the single frame lag activation function (blue), for 10 samples leading to the pressure release point (PRP). (**f**, **l**) Average Doppler intensity variations within the combined mask as a function of time (Doppler frames). Shaded light blue region shows half of the standard deviation on each side of the plot. Green and red circles denote the cuff inflation and deflation points, respectively.

Comparative illustration of the results for the aforementioned cases is displayed in Fig. 1. Figures 1a and g show example B-mode images of scanned area. Obtained Doppler frames after the pressure release point (PRP) are shown in Fig. 1b and h. Binarized correlation masks are presented in Fig. 1c and i. These masks show pixels at which temporal Doppler signals have a correlation larger than 0.5 with the single frame lag activation function. Comparing the two figures, fewer pixels demonstrate a correlated behavior with the activation function (i.e., a rapid hyperemic response to pressure release) in the case of the PAD patient. Figures 1d and j illustrate the lag images for the healthy and affected legs, respectively. Dark blue regions represent lag0 (no lag) pixels where an immediate surge of flow occurs. A larger region is covered by such pixels in Fig. 1d compared to Fig. 1j. Figures 1e and k show the average of the normalized temporal Doppler signals (red line) for all pixels within the correlation masks, as well as the single frame lag activation function (blue line), for 10 frames before PRP up to PRP, exhibiting a sharp increase in the amplitude of the signal at the time of pressure release in correlation with the activation function. The shaded area around the red line in light blue shows half of the standard deviation of variations for all pixels on each side of the line. Figures 1f and l depict the average of the Doppler intensities of the pixels inside the correlation masks at each Doppler frame. These variations demonstrate the hemodynamic response to cuff inflation (green circles) and deflation (red circles). The inflation and deflation frames are chosen as the Doppler frames that are closest in time to the actual inflation and deflation events during the study. The general trend in the responses indicates a decline in flow after inflation and a rise and gradual fall after deflation. A more intense and rapid response to pressure release is observed in Fig. 1f compared to Fig. 1l.

**Figure 2:**
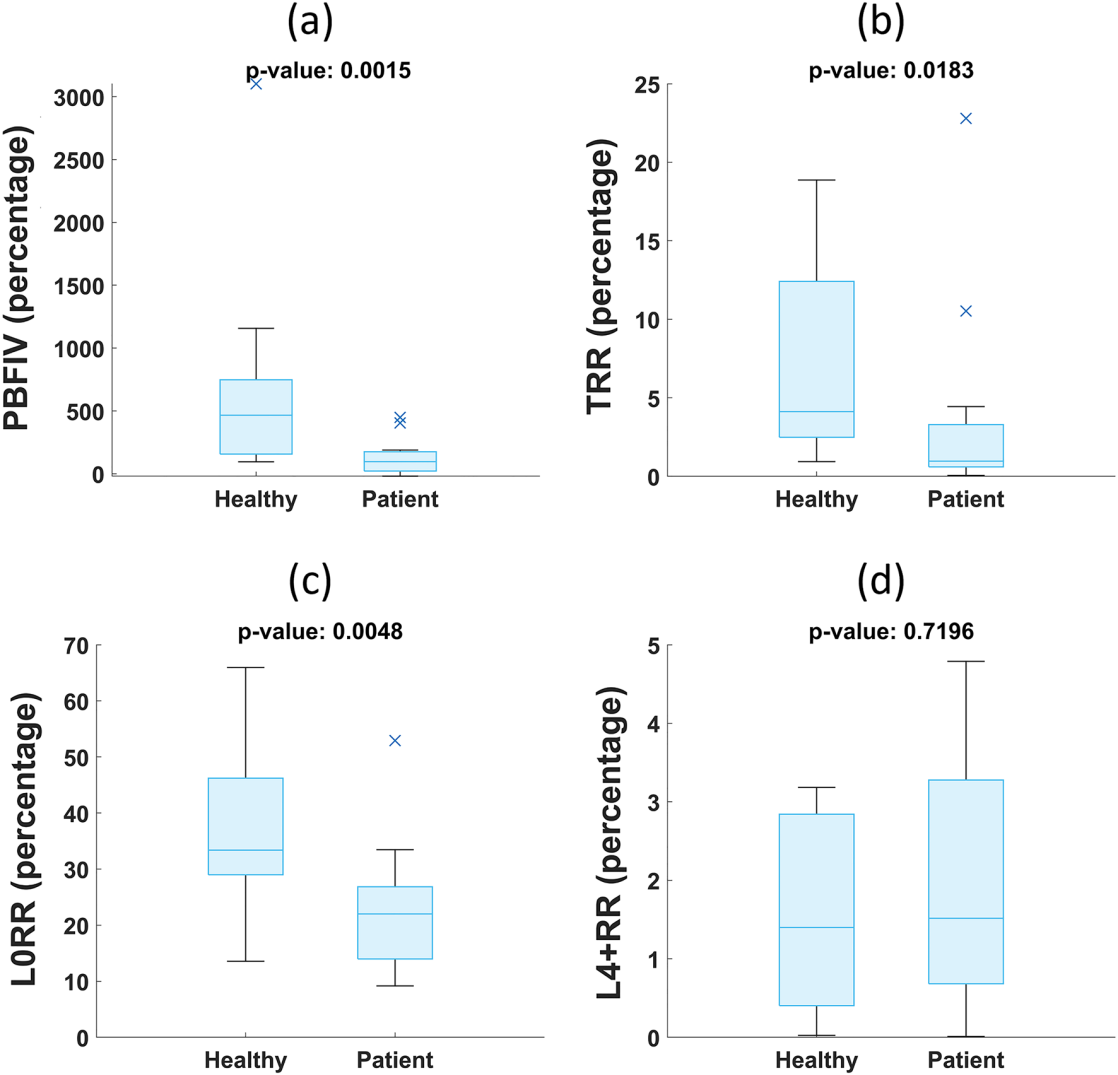
Box-and-whisker plots showing the distributions of the metrics (**a**) post-occlusion to baseline flow intensity variation (PBFIV) (**b**) Total response region (TRR) (**c**) Lag0 response region (L0RR) (**d**) Lag4 (and more) response region (L4 + RR).

A summary of the calculated metric values is presented in Table 1. The corresponding box-and-whisker plots of the distributions of the metrics for the two groups are also illustrated in Fig. 2. The post-occlusion to baseline flow intensity variations (PBFIV) show the net increase in Doppler intensity with respect to the average baseline Doppler intensity. This parameter shows an average of 672 percent increase in Doppler intensity in response to pressure release for the healthy leg compared to 123 percent for the affected legs. Total response region (TRR) represents the percentage of the pixels that have a higher than 60 percent correlation with at least one shifted version of the multiple frame lag activation function. This region constitutes an average of about a 3.61 percent of the entire scanning region for the PAD cases compared to 7.42 percent for the healthy group. Finally, based on the lag images, in the case of affected legs, for nearly 1.99 percent of the scanned area on average, it takes at least four (or more) frames to manifest a hyperemic response (if they do so at all), while this value is about 1.61 percent for the healthy subjects (L4 + RR). On the other hand, an average of about 37.19 percent of the region demonstrates an immediate (lag 0) flow compensation in the case of healthy legs compared to 22.60 percent in the case of affected legs. In total, three out of the four utilized metrics exhibited a significant (*p*-value > 0.05) distributional difference between the two groups.

**Table 1:** Hemodynamic response metrics and their corresponding *p*-values (metrics distributions are presented as mean ± standard deviation).

Metrics	PAD	Healthy	*P*-value
PBFIV* (percentage)	123.02 ± 149.30	672.86 ± 800.94	0.0015
TRR** (percentage)	3.61 ± 6.42	7.42 ± 6.50	0.0183
L0RR*** (percentage)	22.60 ± 11.86	37.19 ± 14.89	0.0048
L4 + RR**** (percentage)	1.99 ± 1.63	1.61 ± 1.22	0.7196

*PBFIV: Post-occlusion to baseline flow intensity variation.

**TRR: Total response region.

***L0RR: Lag0 response region.

****L4 + RR: Lag4 (and more) response region.

## Discussion

In this study, we introduced a contrast-free ultrasound imaging framework for evaluation of blood flow variations in response to pressure cuff occlusion in PAD patients as well as healthy subjects. Post-processing and analysis help in visualization and evaluation of flow changes and quantification metrics can have diagnostic potential.

There exist several studies in the literature for monitoring blood flow and perfusion changes in the lower extremity in patients with PAD using different imaging modalities including MRI^56^, CT^57^, and contrast-enhanced ultrasound^58^ imaging. The potential and significance of the current study lies in its use of simple, inexpensive, and accessible ultrasound imaging without the added burden of injecting contrast agents. This can potentially preclude further discomforts and expenses for the patients, as ultrasound is commonly used for screening purposes.

Implementation of the proposed method on patients with clinically diagnosed PAD (based on ABI evaluation) and healthy subjects suggest that there might be observable differences between the hemodynamic response of affected legs in PAD patients to pressure cuff induced occlusion compared to the legs of healthy individuals. Correlation masks presented in Fig. 1c and i show examples of a healthy leg and an affected leg, respectively, where a larger region exhibits an immediate flow compensation (high correlation with the activation function) in the healthy case.

The normalized average Doppler Intensity variations in the Doppler frames leading up to the point of pressure release (as shown in Fig. 1e and k) exhibit how in both cases blood flow compensation follows the activation function at PRP. The normalized values of Doppler intensities (red curves), however, are slightly lower in the healthy case. This is expected, as these values are normalized by the maximum intensity at PRP, and therefore a lower normalized value is equivalent to a higher increase in blood flow as a response to pressure release.

Doppler intensity variations as shown in Fig. 1f and l illustrate how in both cases there is a general decline in the amount of blood flow as a response to pressure cuff inflation (points after the green circles). Similarly, there is a rapid increase in the Doppler intensity following pressure release points (red circles). This hyperemic response to cuff occlusion has been observed in other studies^54,59^. Nonetheless, several differences are noticeable between the two intensity profiles. As reported in Table 1, the average relative post-occlusion increase in Doppler intensities compared to baseline for PAD patients is about 123 percent, while this value is 672 percent for the healthy subjects. This is in keeping with several studies suggesting reduced reactive response in leg blood flow for PAD patients^60^. Another observation in the Doppler intensity profiles is the slower decline in post-occlusion response in the case of the PAD patient. Additionally, peak Doppler intensity seems to occur a few frames after pressure release (as opposed to the healthy case, where the peak appears immediately following pressure release). This phenomenon has been reported in the literature where PAD subjects generally exhibit a faster perfusion increase as well as a more rapid drop in perfusion post occlusion^23^.

The delayed response in the case of PAD patients is also quantified via the percentage of no lag pixels (L0RR) and the pixels with a lag of four frames and more (L4 + RR). This is in keeping with observations in the literature where the time to peak response in PAD patients is reportedly significantly higher^55^.

There are some limitations associated with the current study. Firstly, the number of subjects included in the analysis for this study was limited. In the future, we plan to study on a large patient population, PAD patients as well as healthy volunteers, to evaluate the performance of the introduced technique in differentiating between the two groups based on the quantification metrics. We also plan to incorporate exercise as part of our setup to evaluate the combined post-exercise and post-occlusion responses for enhanced differentiation between affected and unaffected lower limbs.

## Methods

### In vivo study

To evaluate the efficacy of our methods, imaging studies were conducted for 14 patients with clinical diagnosis of PAD (7 male, 7 female), and 8 healthy volunteers (1 male, 7 female). Age distribution of the patients and healthy subjects was 65.9 ± 16.3 and 64.1 ± 2.8, respectively (mean ± standard deviation). This study was approved by the institutional review board at the Mayo Clinic (IRB#: 19-002559) and was in compliance with the Health Insurance Portability and Accountability Act. All the research was performed in accordance with relevant guidelines/regulations and followed the Declaration of Helsinki. A written IRB approved informed consent form was signed by each participant prior to the study. Patients were recruited based on their clinical ABI examination. Recruitment for the healthy group involved aiming for non-smoker individuals with no history of diabetes or relevant underlying diseases. During the study, both legs of the subjects were scanned. For final data analysis, data from legs of patients with normal ABI, as well as poor data acquisitions were excluded. After exclusions, data from a total of 13 legs with abnormal ABI were compared to data from 13 legs of healthy individuals.

### Data acquisition

Data acquisition was performed using a linear array L11-4v probe (with a center frequency of 7.24 MHz) attached to a Verasonics Vantage 256 ultrasound research system (Verasonics Inc., Kirkland, WA, USA). Ultrafast ultrasound imaging was implemented through coherent compounding of plane wave transmissions at five different insonification angles equally spaced within the [− 5.5° +5.5°] range. Each transmission sequence involved acquiring 500 IQ data frames at a frame rate of 2 kHz and was repeated every 2 s to monitor the flow response over time.

### Experimental setup

**Figure 3:**
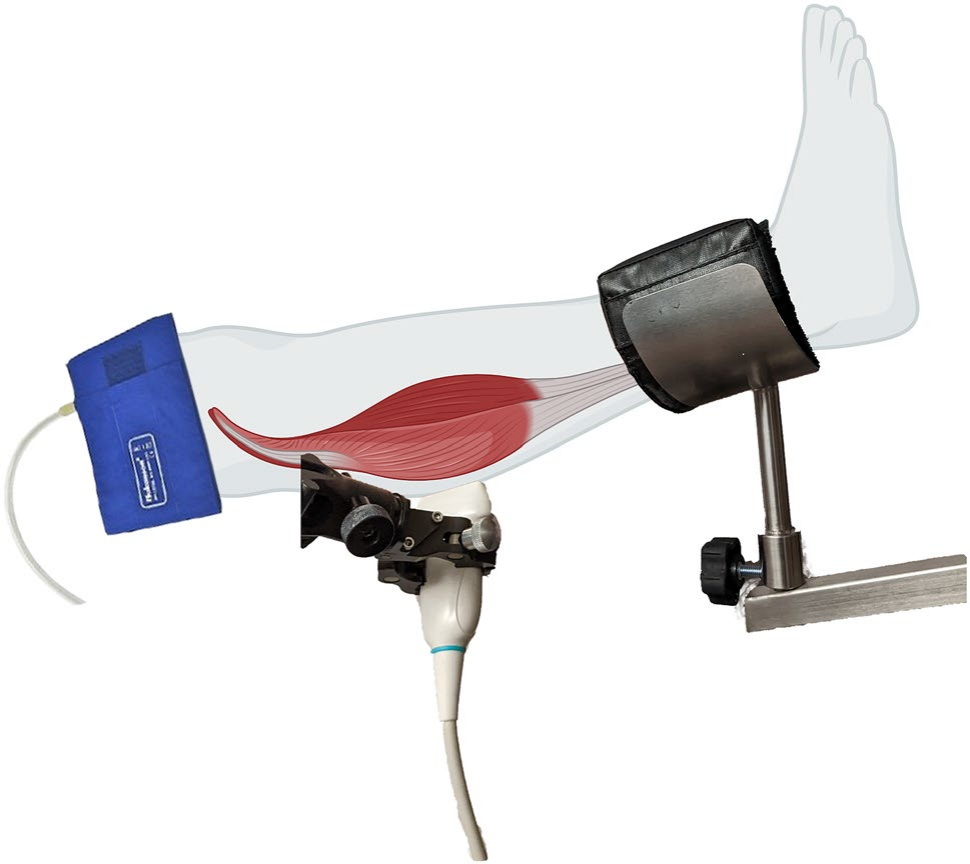
The experimental setup for data acquisition. An ultrasound probe is fixed using a mechanical arm and is attached to the calf muscle for the duration of the study. The leg is placed on a leg prepper and a pressure cuff is wrapped around the thigh.

**Figure 4:**
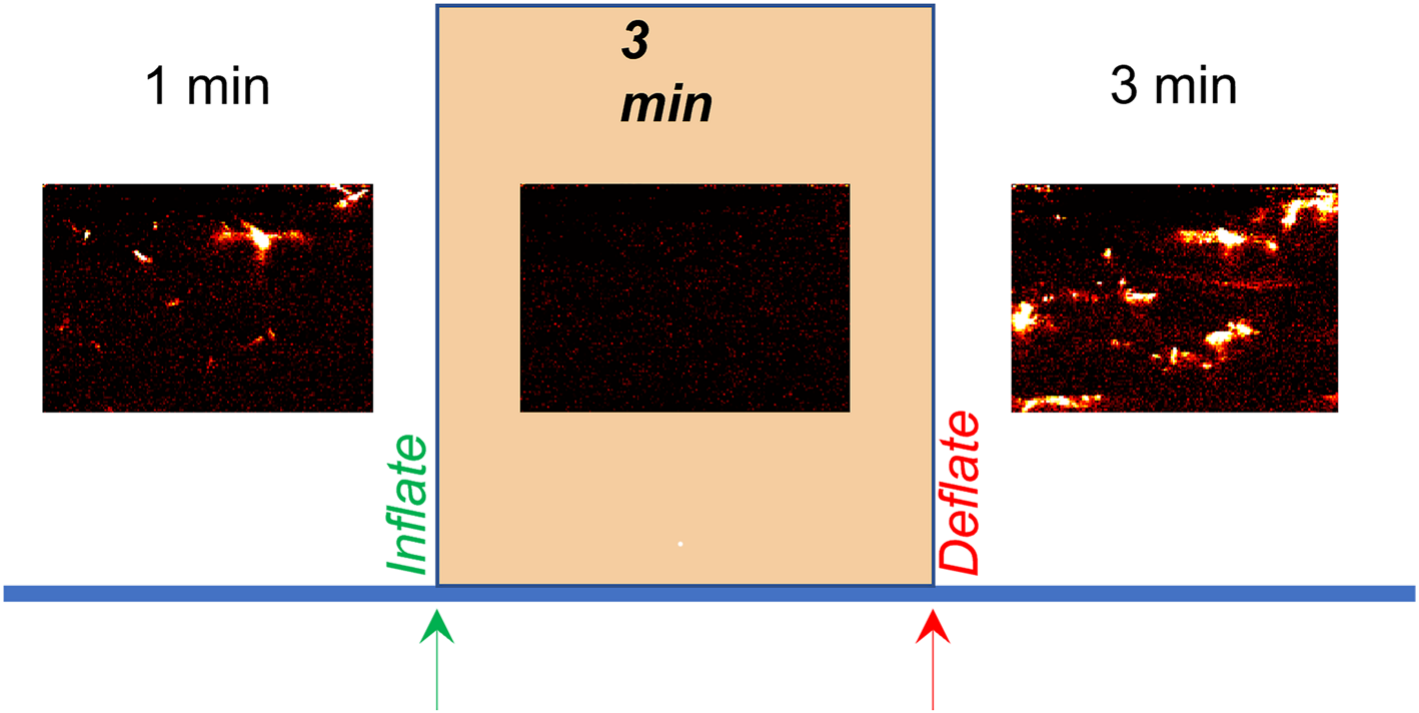
The timeline of the data acquisition procedure, consisting of a minute of baseline acquisition, three minutes of pressure cuff occlusion, and three minutes of post-occlusion acquisition. Example Doppler images are shown within each section of the timeline.

With subjects in the supine position, the targeted leg was placed on a stand-alone leg prepper. The ultrasound probe was attached to the subject’s calf muscle for imaging and was secured in place using a multi-joint mechanical arm. A pressure cuff was wrapped around the subject’s thigh and an automatic cuff inflation device (D. E. Hokanson Inc., Bellevue, WA, USA) was used for rapid inflation of the cuff. A schematic illustration of the setup is shown in Fig. 3. Ultrasound data was continuously collected for 7 min per each leg. The setup consisted of one minute of baseline data acquisition, followed by three minutes of pressure-induced occlusion and three minutes of post-occlusion data acquisition. Figure 4 depicts a schematic of the timeline of the acquisition setup, as well as examples of Doppler images corresponding to each portion of the study.

### Doppler image generation

The 500 IQ data frames acquired during each transmission sequence constitute an ensemble of ultrasound images that was used to generate a Doppler image visualizing blood flow intensities at a given point in time. These frames were first reshaped into a spatiotemporal/Casorati data matrix. Next, a singular value decomposition (SVD) filter was applied to the data to remove the low-rank tissue clutter. In this study, the SVD threshold for the separation of clutter and blood subspaces was chosen empirically and was set to the constant value of 50 for all the Doppler frames (no upper threshold for noise removal). Subsequently, the clutter filtered data frame ensemble underwent a temporal coherent integration to generate the Doppler image.

**Figure 5:**
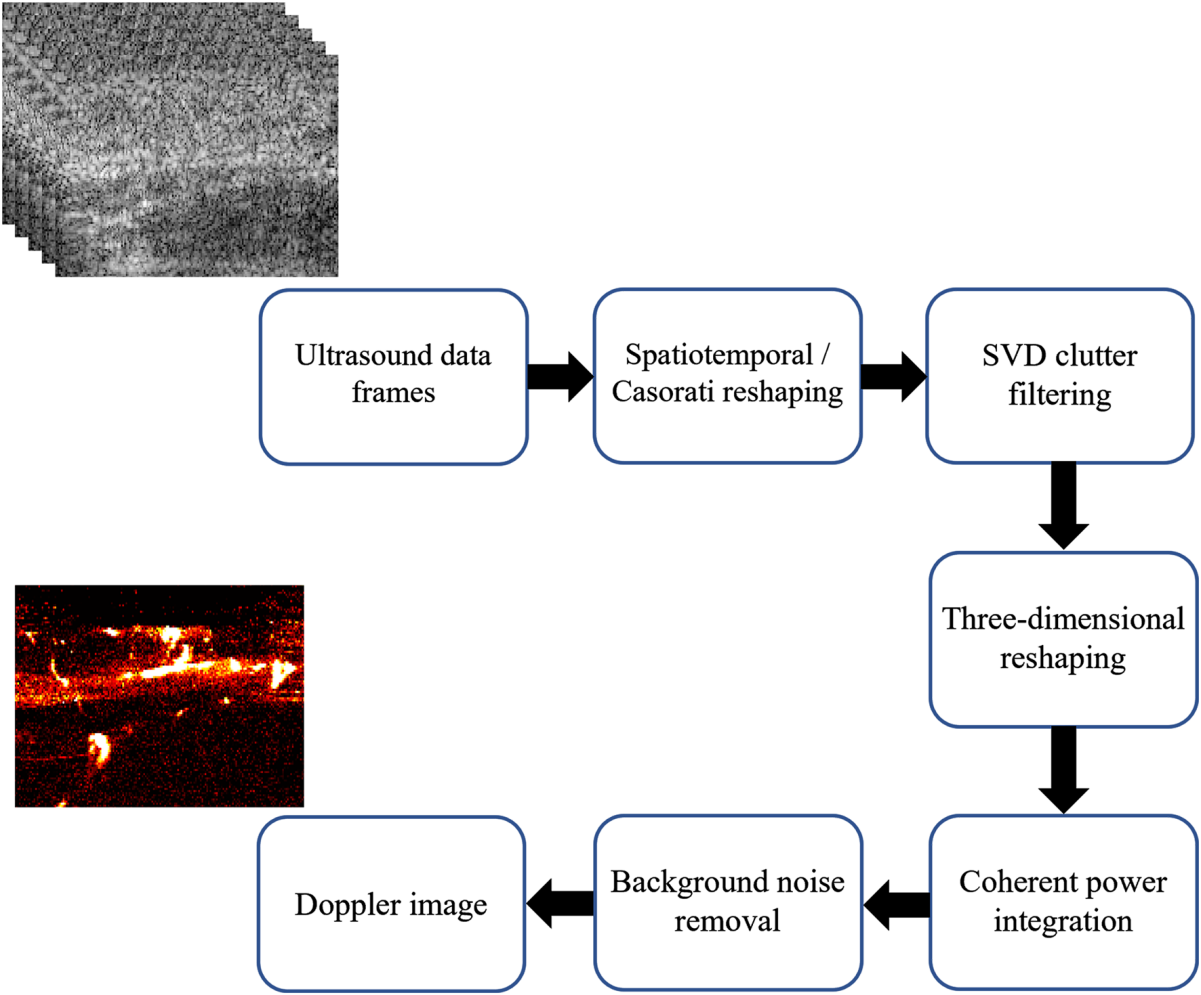
The flowchart of Doppler image generation algorithm.

The resulting Doppler image was composed of signals generated by blood flow as well as noise. The background noise profile (mainly induced by the time gain compensation (TGC) settings) can be estimated and compensated for, through various means^61,62^. In this work, we used a Doppler image generated from an open-air transmission to estimate this profile. Since no significant echo signal was expected in such a transmission, the resulting image would be a good approximation of the TGC-induced noise pattern. Considering the TGC-induced noise was removed after the implementation of SVD, it did not have a direct influence on our choice of the SVD threshold. We then subtracted this noise profile from each of the Doppler images to generate a final flow image. We refer to each of these images as a Doppler frame. Figure 5 illustrates different stages of generating these Doppler frames.

### Hemodynamic analysis

The generated Doppler images illustrate the hemodynamic variations in the calf muscle. These variations can be quantified as a function of time, in a time series with a sampling period of 2 s. To monitor the hyperemic response to the pressure induced occlusion, we define a single lag activation step function to represent the onset of the stimulation (pressure release) at the “Deflate” (pressure release) point. We then correlate the Doppler intensity variations from 10 Doppler frames before, up until one frame after the point of pressure release. This results in a correlation value for each pixel in the Doppler image showing its consistency with the expect hyperemic response, together constituting a correlation map after pressure release. Using a threshold on these maps, a binarized image is generated depicting all the pixels that exhibited a compensatory flow response, including muscle perfusion. This threshold was set to 0.5 in this study to strike a balance between the inclusion of slow responding flow elements (such as muscle perfusion signals) and the inclusion of noise. We refer to these binarized images as correlation masks. These correlation masks are utilized to monitor Doppler intensity alterations, by averaging the masked Doppler signal magnitude as a function of Doppler frame sample time for the duration of the study. These temporal intensity profiles can then be used to estimate post-occlusion Doppler intensity variations with respect to the baseline. Therefore, we define a metric called “post-occlusion to baseline flow intensity variation” (PBFIV) as the percentage-wise ratio of the difference between average post-occlusion and average baseline flow intensities over the average baseline intensity:

1 $$PBFIV \left( \% \right) = \frac{{I_{{Post - occ - 5frames_{mean} }} - I_{{Baseline_{mean} }} }}{{I_{{Baseline_{mean} }} }} \times 100$$

$I_{{Post - occ - 5frames_{mean} }}$ is the average of Doppler intensities in the first five post-occlusion Doppler frames, and ${I}_{{Baseline}_{mean}}$ is the average of Doppler intensities in all of the baseline Doppler frames. Additionally, by defining a multiple (in this case 4) frame lag activation function we evaluate the delay in post-occlusion response. By cross-correlating the Doppler intensity variations with shifted versions of this activation function and finding the shifts that result in maximum correlations, we determine how many post-occlusion frames it takes for each pixel to exhibit a compensatory flow response. Subsequently, we generate maximum correlation maps showing all the pixels that exhibit a hyperemic response and through binarization (threshold of 0.6) and pixel count, estimate the total response region (TRR):

2 $$TRR \left( \% \right) = \frac{{N_{MaxCorr} }}{{A_{Image} }} \times 100$$

$N_{MaxCorr}$ is the number of nonzero pixels in the binarized maximum correlation map (Fig. 1c and i), and $A_{Image}$ is the total number of pixels (area) of the maximum correlation map/image.

Moreover, by creating maps of post-occlusion response delay (referred to as lag images) we estimate the lag-specific response regions. Lag0 response region (L0RR) corresponds to the density of pixels exhibiting an immediate increase flow following pressure release, and Lag4 (and more) response region (L4 + RR) represents the pixels for which at least 4 frames are required to manifest a compensatory response (flow increase):

3 $$L0RR \left( \% \right) = \frac{{N_{Lag0} }}{{A_{Image} }} \times 100$$

4 $$L4 + RR \left( \% \right) = \frac{{N_{Lag4 + } }}{{A_{Image} }} \times 100$$

${N}_{Lag0}$ is the number of pixels corresponding to Lag0 and ${N}_{Lag4+}$ is the number of pixels corresponding to Lag4 (and more) in the lag image (Fig. 1d and j), and ${A}_{Image}$ is the total number of pixels (area) of the lag image. Consequently, PBFIV quantifies the relative increase in blood flow in response to cuff occlusion; TRR approximates the density of the pixels that exhibit a hyperemic response within a few frames post-occlusion; L0RR represents the relative size of the region where the quickest measurable response to cuff occlusion occurs; and L4 + RR shows the extent of the region where the hyperemic response (if any) takes at least 4 Doppler frames to appear.

**Figure 6:**
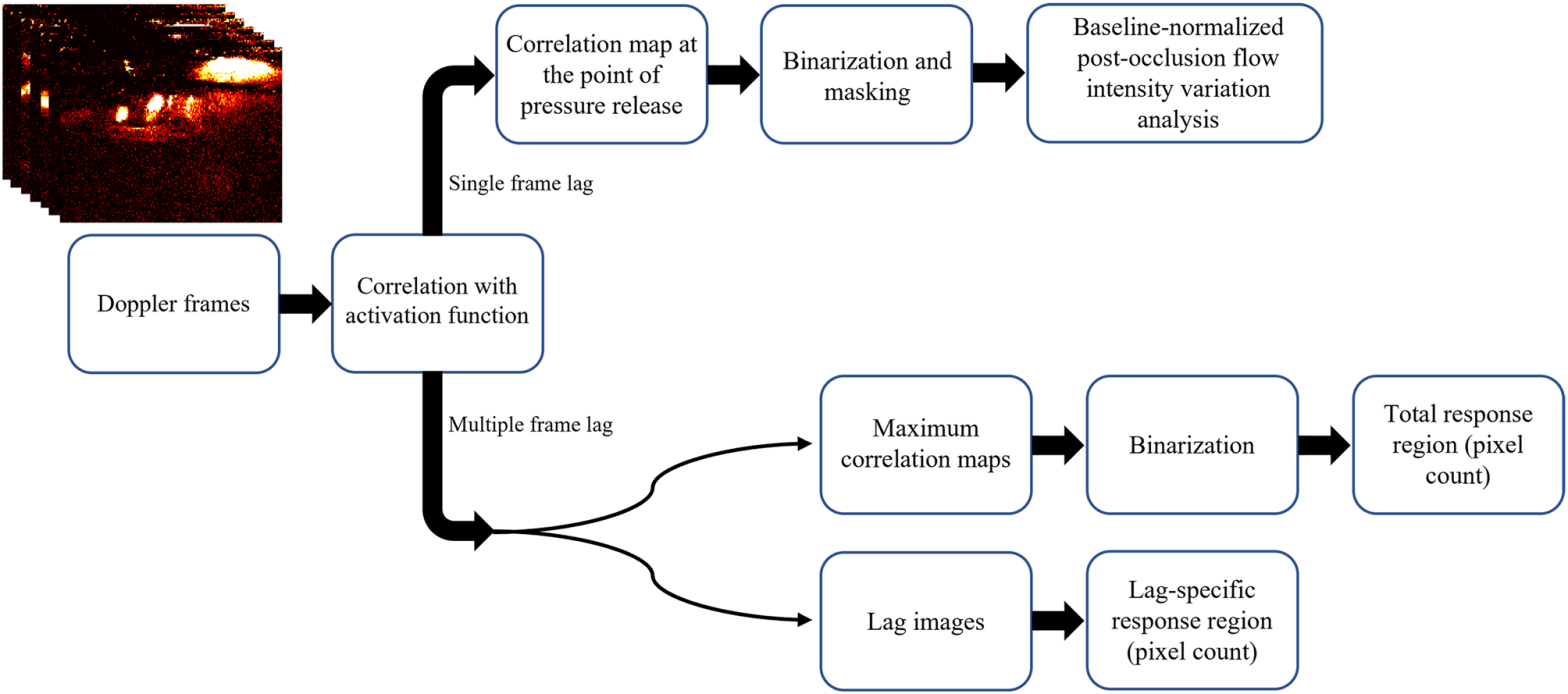
The flowchart of hemodynamic response analysis.

**Figure 7:**
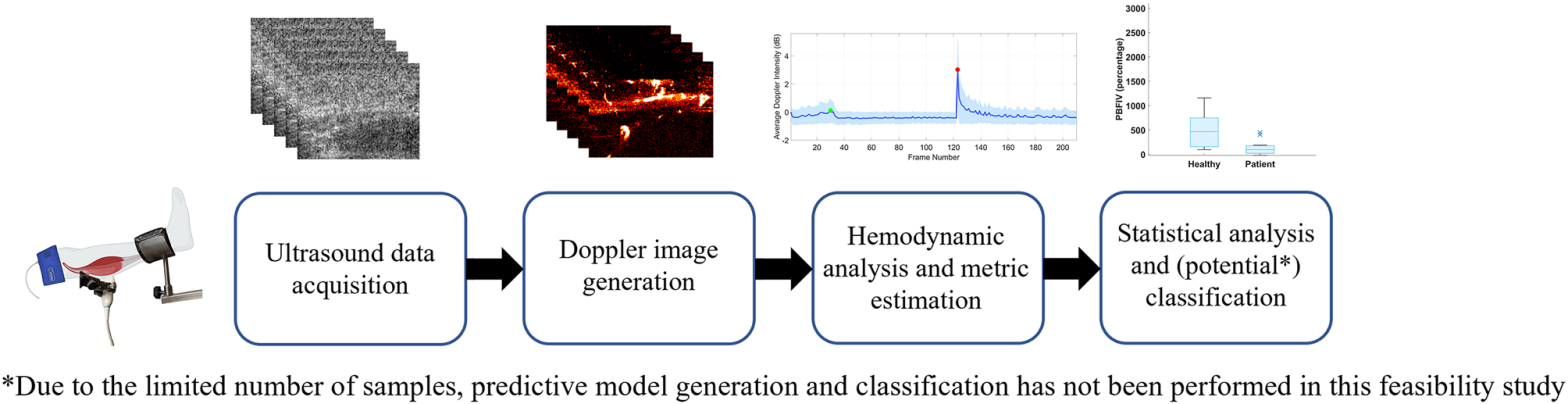
The methodology flowchart.

Figure 6 shows different stages of this process. Figure 7 contains a flowchart of the entire method, from data acquisition to metric estimation for potential diagnostic applications.

### Statistical analysis

The previously defined metrics were calculated for the two groups (patient legs with abnormal ABI, and healthy legs). A two-sided Wilcoxon rank sum test was implemented on the distributions of these metrics to evaluate the differences between the two groups and the corresponding p-value was computed for each metric. Differences in metrics with p-values less than 0.05 were considered to be statistically significant. Data processing and statistical analyses were performed in MATLAB R2022b (Mathworks Inc., Natick, MA, USA).

## Conclusion

In this paper, we presented a contrast-free ultrasound imaging technique for monitoring the hemodynamic response to pressure cuff induced occlusion in the lower extremity of PAD patients. Metrics were defined to analyze and quantify the variations in blood flow in a baseline state, and after pressure release. These quantification metrics can be utilized to differentiate between affected and unaffected legs and potentially help with more accurate diagnosis of PAD patients before the need for more costly cross-sectional imaging approaches arises. In our future studies, we plan to recruit larger numbers of patients and perform statistical analyses on the proposed metrics to evaluate their discriminatory potential.

## Data Availability

The data that support the findings of this study are available from the corresponding author upon reasonable request. The requested data may include figures that have associated raw data. Because the study was conducted on human volunteers, the release of patient data may be restricted by Mayo policy and needs special request. The request can be sent to: Karen A. Hartman, MSN, CHRC|Administrator—Research Compliance| Integrity and Compliance Office | Assistant Professor of Health Care Administration, Mayo Clinic College of Medicine & Science | 507-538-5238 | Administrative Assistant: 507-266-6286 | hartman.karen@mayo.edu Mayo Clinic | 200 First Street SW | Rochester, MN 55905 | mayoclinic.org.m We do not have publicly available Accession codes, unique identifiers, or web links.
